# Diagnostic accuracy of imaging modalities for detection of spinal metastases: a systematic review and meta-analysis

**DOI:** 10.1007/s12094-024-03765-1

**Published:** 2024-10-29

**Authors:** Netanja I. Harlianto, Simone van der Star, Britt B. M. Suelmann, Pim A. de Jong, Jorrit-Jan Verlaan, Wouter Foppen

**Affiliations:** 1 Department of Orthopedic Surgery, University Medical Center Utrecht & Utrecht University, Utrecht, The Netherlands; 2 Department of Radiology and Nuclear Medicine, University Medical Center Utrecht & Utrecht University, Utrecht, The Netherlands; 3 Department of Radiation Oncology, University Medical Center Utrecht & Utrecht University, Utrecht, The Netherlands; 4 Department of Medical Oncology, University Medical Center Utrecht & University Utrecht, Utrecht, The Netherlands

**Keywords:** Spinal metastases, Oncology, Diagnostic meta-analysis, Computed tomography, Magnetic resonance imaging, Positron emission tomography

## Abstract

**Purpose:**

Detecting spinal metastases is highly relevant in patients with oncological disorders as it can affect the staging and treatment of their disease. We aimed to evaluate the diagnostic performance of computed tomography (CT), magnetic resonance imaging (MRI), FDG positron emission tomography (PET)/CT, bone scintigraphy (BS), and single-photon emission computed tomography (SPECT) for spinal metastases detection.

**Methods:**

Medline, EMBASE, and Web of Science were systematically searched until March 2024 for diagnostic accuracy studies on spinal metastases detection (PROSPERO-registration: CRD42024540139). Data extraction and quality assessment using the QUADAS-2 tool were performed by two independent reviewers. Using bivariate random effects modeling, pooled sensitivities, specificities, and diagnostic odds ratios (DOR) were calculated, and hierarchical summary operating curves were constructed.

**Results:**

Twenty-five studies (49 datasets), encompassing 3102 patients were included. Per-patient pooled sensitivities of CT, MRI, PET/CT, BS and SPECT were 70%, 93%, 82%, 75%, and 84%, respectively. Pooled specificities were 74%, 85%, 75%, 92%, and 81%, respectively. Per-lesion pooled sensitivities of CT, MRI, PET/CT, BS and SPECT were 76%, 91%, 92%, 77%, and 92%, respectively. Pooled specificities were 91%, 94%, 85%, 52%, and 86%, respectively. MRI had the highest DOR in per patient and lesion analyses.

**Conclusion:**

MRI had highest diagnostic accuracy for spinal metastases detection on patient and lesion level, suggesting a broader use in addition to the routine staging CT, at least in patients at high risk and where the detection of a spinal metastasis could alter therapy decisions. Herein, results should be considered with the limitations of each modality.

**Supplementary Information:**

The online version contains supplementary material available at 10.1007/s12094-024-03765-1.

## Introduction

Spinal metastases account for up to 40% of distant metastases sites in oncological patients with metastatic disease [1, 2]. The prevalence of spinal metastases is expected to rise as a result of the growing cancer burden worldwide and the improved efficacy of cancer treatments, which allow patients to live longer [3]. Breast, prostate, and lung cancer most commonly metastasize to the spine [3]. Patients with symptomatic spinal metastases frequently experience severe neck or back pain, and are at risk for pathological fractures with vertebral body collapse, and the development of neurologic deficits [4]. Timely treatment of spinal metastases is important in order to maintain quality of life in this population, which often consists of surgery and radiotherapy treatment [5, 6]. Herein, imaging is essential for the early detection of spinal metastases to ensure appropriate treatment and a reduction in patient morbidity as a result of pain or spinal complications.

Conventional imaging modalities such as computed tomography (CT) and magnetic resonance imaging (MRI) are important techniques for diagnosing and staging malignancies, and they are commonly used in clinical practice due to their widespread availability [7, 8]. These modalities, however, have their shortcomings, such as the inability to detect small lesions (on CT) or incorrect interpretation of metastasis as benign lesions, which are very common in the spine. Functional nuclear imaging is able to overcome these limitations to a certain extent, which most commonly includes positron emission tomography (PET)/CT, bone scintigraphy (BS) and single photon emission computed tomography (SPECT). PET/CT provides insight into elevated metabolic activity of tumors and anatomical details, facilitating the early identification of lesions and response to treatment evaluation [9]. However, not all tumor subtypes are seen on PET/CT [9]. Tumor activity can also be visualized using planar imaging with BS, which has shown good sensitivity for (sclerotic/blastic) bony metastases, but lacks anatomical markers posing challenges to precisely localize sites suspected of malignancy [10]. This shortcoming is overcome with SPECT which combines both functional imaging and anatomical location [11–13]. Advanced imaging, however, is limited by its availability and costs. Many studies have been performed to investigate the diagnostic accuracy of these conventional and advanced imaging modalities in spinal metastases, including more recent publications [14–17].

In the present systematic review and meta-analysis, we assess the diagnostic value and reevaluate the state of the available evidence for CT, MRI, PET/CT, BS, and SPECT in the detection of spinal metastases. In addition, the clinical applicability of these results are contextualized in the setting of routine cancer staging.

## Methods

### Data sources and study selection

This diagnostic meta-analysis and systematic review was conducted according to the Preferred Reporting Items for a Systematic Review and Meta-analysis of Diagnostic Test Accuracy Studies [18] (Appendix A) and was prospectively registered in PROSPERO (CRD42024540139). A systematic literature search was performed in Medline, EMBASE, and Web of Science until March 15, 2024 for articles assessing the diagnostic performance of CT, MRI, PET/CT, SPECT, and BS for spinal metastasis detection. A search syntax was constructed using a combination of the terms “spinal metastases”, “CT”, “MRI”, “PET”, “PET/CT”, “BS”, “diagnostic test accuracy measures” with relevant synonyms. The full search syntax can be accessed in Appendix B. No language restrictions were applied, and the reference lists of included articles were screened to identify additional articles not included in the original search. In addition, authors were contacted for additional clarification and/or data.

Title and abstracts were independently screened by two investigators (N.I.H. and S.v.d.S.), with disagreements resolved with a third reviewer (W.F.). Inclusion criteria were (1) articles describing 10 or more patients; (2) CT, MRI, PET/CT, BS or SPECT for spinal metastases detection; (3) reference standard available, which included confirmation by imaging, pathology, and/or clinical and imaging follow-up; (4) Patient and/or lesion data available of the true-positive (TP), true-negative (TN), false-positive (FP) and false-negative (FN). TP/TN/FP/FN results were derived from marginal totals or sensitivity and specificity if not specifically stated using formulas of sensitivity (TP/(TP + FN)) and specificity (TN/(TN + FP)). If these results were not retrievable, we contacted authors for clarification or additional data. Exclusion criteria were case reports, letters, editorials, review articles, animal studies.

### Data extraction and quality assessment

Data extraction was performed independently by two investigators (N.I.H. and S.v.d.S.) using a predetermined data form. A third reviewer (P.A.d.J.) was consulted in the case of disagreements between investigators. The following variables were extracted: author, year, study design, study period, diagnostic imaging modality, single or multicenter study, tumor type, level of analysis (patient or lesion), and reference standard. Quality assessment was independently performed with disagreements discussed by consensus using the revised tool for the quality assessment of diagnostic accuracy studies (QUADAS-2), which covers the domains patient selection, index test, reference standard, and flow and timing. Each of the four domains was scored on risk of bias as “low”, “high” or “unclear” [19]. Furthermore, applicability concerns were evaluated as ‘low’, ‘high’ or ‘unclear’ with regards to the first three domains [19].

### Statistical analysis

Pooled estimates of sensitivity, specificity, and diagnostic odds ratios (DOR) with corresponding 95% confidence intervals (95% CI) were obtained using bivariate random effects modeling. DOR is a diagnostic performance metric that combines specificity and sensitivity, for which a DOR of 1 indicates no discriminatory power, and an increasing DOR indicates the test’s ability to distinguish between patients with and without the target disorder (spinal metastases) [20]. Using the summary receiver operating characteristic (SROC) model, summary receiver operating characteristic curves were constructed for each imaging modality on patient and on lesion level. The Higgin’s and Thompson *I*^*2*^ statistic was utilized to quantify heterogeneity [21]. Low heterogeneity was defined as *I*^*2*^ < 50%, moderate heterogeneity as *I*^*2*^ = 50–75%, and high heterogeneity as *I*^*2*^ > 75%. Publication bias was assessed using Deek’s Funnel plots [22]. Statistical analysis was performed using R, version 4.1.3 (R Foundation for Statistical Computing) using the *meta* and *mada* package. A *p*-value < 0.05 was considered as statistical significance.

## Results

### Study selection

**Fig. 1:**
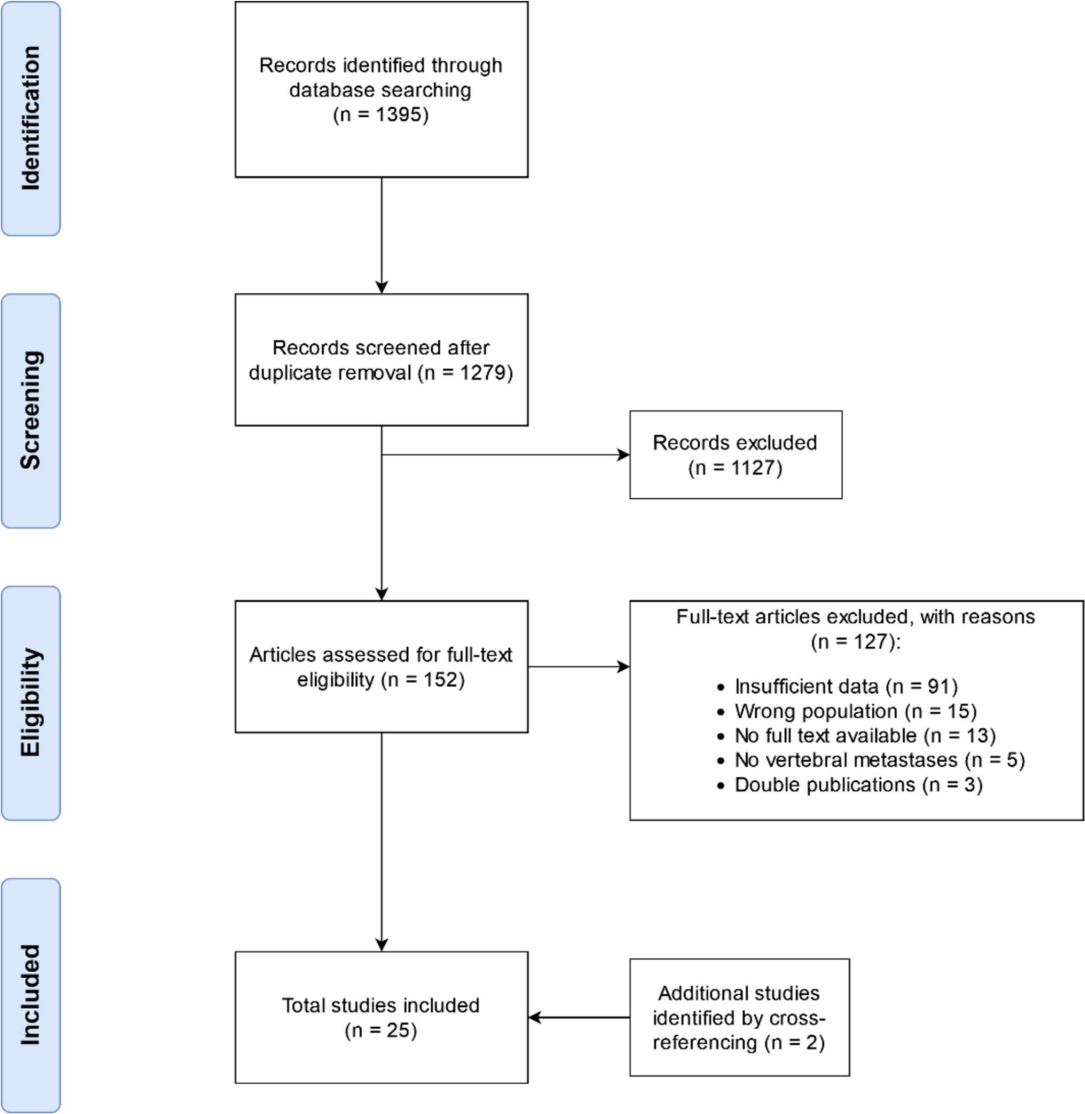
PRISMA flowchart of study selection

A total of 1395 articles were identified from electronic database searching after excluding duplicates. Title and abstract screening was performed for 1279 articles, for which 152 articles were assessed for full-text eligibility. Finally, after excluding 127 articles with reason, 25 articles encompassing 49 unique imaging datasets in 3102 patients were included in the current meta-analysis [14–16, 23–44]. The flowchart of the study selection process is provided in Fig. 1.

### Study characteristics

Baseline study characteristics are provided in Table 1. The median study population of all included studies was 66 (interquartile range: 40–121, range: 21–410), the median percentage of males 52% (interquartile range: 44–61%), and the reported mean ages ranged from 46 to 69 years. All included studies were single center studies, and the majority were retrospective in design (*n* = 19), with three studies being prospective, and three studies not specified. Reference standards used included imaging (*n* = 10), imaging, pathological confirmation, and follow-up (*n* = 6), imaging and follow-up (*n* = 6), and pathological confirmation (*n* = 3). Stratified by tumor type, the majority of studies had mixed primary tumor types (*n* = 17), followed by breast cancer (*n* = 5), prostate cancer (*n* = 2), and lung cancer (*n* = 1). Two articles were published between 1990 and 1999, 7 articles between 2000 and 2009, and 16 articles between 2010 and 2023.

**Table 1:** Baseline characteristics of included studies

Author	Year	Country	Period	Patients	% male	Primary tumor	Design	Reference	Modality
Cristo Santos [23]	2023	Portugal	2014–2020	410	0	Breast	Prospective cohort	Imaging	FDG-PET, BS
Panagiotidis [15]	2023	UK	2010–2021	66	0	Breast	Retrospective cohort	Imaging, FU	MRI
Zarad [24]	2023	Egypt	2021–2021	40	55	Mixed	NS	Imaging	MRI
Qin [17]	2022	China	2019–2021	102	NS	Mixed	Retrospective cohort	Pathology	CT, SPECT
Jung [25]	2021	Korea	2016–2018	44	64	Mixed	Retrospective case–control	Pathology, imaging, FU	MRI
Liu [16]	2020	China	2010–2017	121	51	Mixed	Retrospective cohort	Pathology	CT, MRI, SPECT
Abdullayev [26]	2019	Germany	2017–2017	21	29	Mixed	Retrospective cohort	Imaging	CT
Mavriopoulou [27]	2018	Germany	2011–2014	257	0	Breast	Prospective cohort	Imaging, FU	BS, SPECT
Maeder [28]	2018	Switzerland	2014–2016	121	52	Mixed	Retrospective cohort	Imaging	MRI
Hahn [42]	2018	Korea	2014–2016	33	61	Mixed	Retrospective cohort	Imaging	MRI
Iwano [29]	2017	Japan	2006–2014	30	80	Lung	Retrospective case–control	Imaging	CT
Park [30]	2017	Korea	2005–2010	481	50	Mixed	Retrospective cohort	Imaging	BS
Lange [31]	2016	Denmark	2011–2013	395	55	Mixed	Retrospective cohort	Pathology	CT, MRI, FDG-PET, BS
Zidan [32]	2014	Egypt	2012–2013	56	55	Mixed	Prospective cohort	Pathology, imaging, FU	MRI
Wafaie [33]	2013	Egypt	NS	22	41	Mixed	NS	Imaging, FU	CT, MRI, FDG-PET
Uchida [34]	2013	Japan	2005–2008	227	53	Mixed	Retrospective cohort	Pathology, imaging, FU	FDG-PET, BS
Venkitaraman [35]	2009	UK	2001–2005	99	100	Prostate	Retrospective cohort	Imaging, FU	MRI, BS
Buhmann [36]	2009	Germany	2002–2006	41	44	Mixed	Retrospective cohort	Pathology, imaging, FU	CT, MRI
Nozaki [37]	2008	Japan	NS	39	100	Prostate	NS	Imaging	BS, SPECT
Oh [38]	2005	Korea	2004–2005	46	NS	Mixed	Retrospective cohort	Imaging	FDG-PET
Ohno [39]	2003	Japan	NS	48	52	Mixed	Retrospective cohort	Pathology, imaging, FU	MRI
Altehoefer [40]	2001	Germany	1992–1999	81	NS	Breast	Retrospective cohort	Pathology, imaging, FU	MRI, BS
Savelli [41]	2001	Italy	1997–2000	118	NS	Mixed	Retrospective cohort	Imaging, FU	SPECT
Han [44]	1998	Hong Kong	1996–1997	174	45	Mixed	Retrospective cohort	Imaging, FU	BS, SPECT
Petrén-Mallmin [43]	1993	Sweden	NS	30	100	Breast	Retrospective cohort	Imaging	CT, MRI, BS

*NS* Not specified, *FU* Follow-up, *CT* Computed tomography, *MRI*:Magnetic resonance imaging, *PET* Positron emission tomography, *BS* Bone scintigraphy, *SPECT* Single photon emission computed tomography, *FDG* Fluorodeoxyglucose;

### Quality assessment

**Fig. 2:**
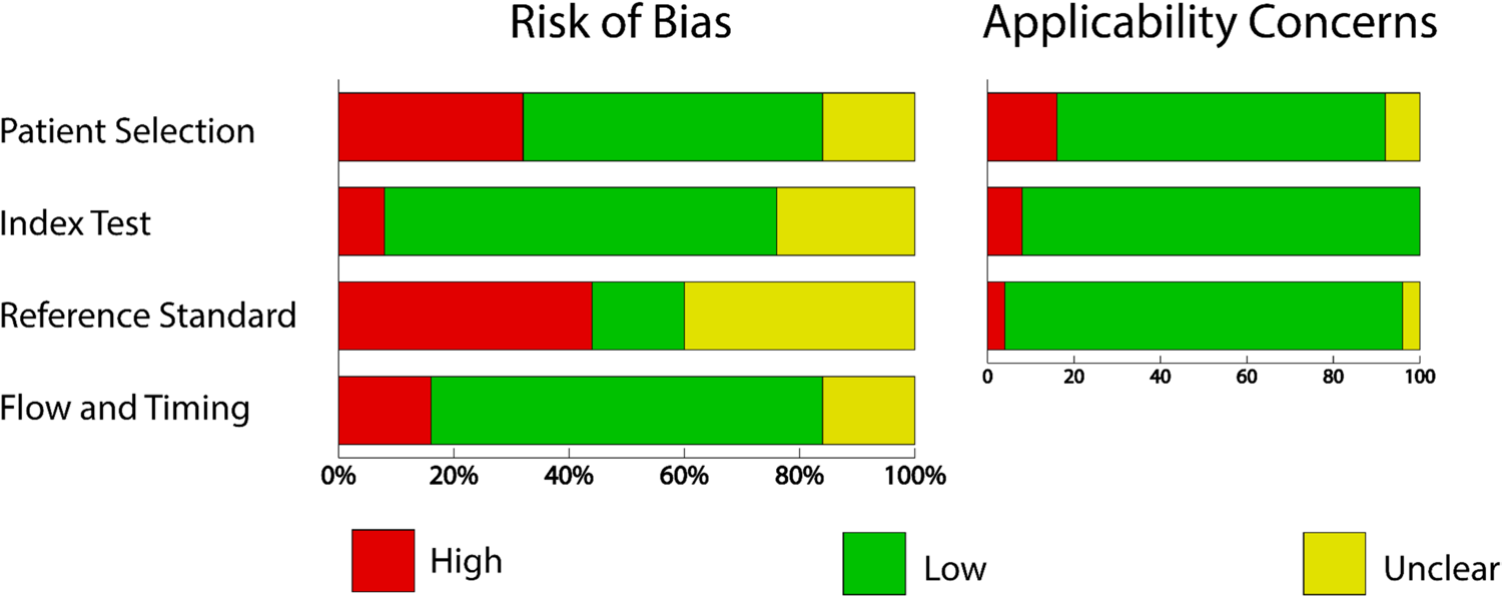
Quality assessment summary

QUADAS-2 assessment results are shown in Fig. 2. Regarding the patient selection domain, high risk was attributed to inappropriate exclusions or an inappropriate extended interval between imaging modalities. For the reference standard domain, a large portion of studies evaluated the reference standard with knowledge of the index test, or this aspect was not clearly specified. The majority of high risk of bias for the domain flow and timing was due to inappropriate time between index test and reference standard. Individual study quality assessment for risk of bias and applicability concerns are provided in Appendix C.

### Diagnostic performance of imaging modalities: patient level

On a per patient level, 5 datasets were available for analysis for CT, 9 for MRI, 2 for FDG-PET/CT, 4 for BS, and 3 for SPECT (Table 2). For CT, the pooled sensitivity and specificity were 70.4% (95%CI 61.5–78.0%) and 73.8% (95%CI 61.8–83.1%), respectively. For MRI, the pooled sensitivity and specificity were 92.6% (95%CI 86.8–96.0%) and 84.7% (95%CI 66.6–93.9%), respectively. Pooled sensitivities and specificities for FDG PET/CT were 81.8% (95%CI 64.9–91.7%) and 74.7% (95%CI 56.9–86.9%), respectively.

**Table 2:** Summary of datasets for each imaging modality

Imaging modality	No. of datasets	No. of patients/lesions	Pooled Sensitivity (95% CI)	Pooled Specificity (95% CI)	Diagnostic odds ratio (95% CI)
Per patient analysis					
CT	5	547	70.4% (61.5–78.0%)	73.8% (61.8–83.1%)	9.7 (6.6–14.3)
MRI	9	631	92.6% (86.8–96.0%)	84.7% (66.6–93.9%)	26.6 (14.3–49.4)
PET/CT	2	68	81.8% (64.9–91.7%)	74.7% (56.9–86.9%)	8.8 (2.8–28.9)
BS	4	690	75.2% (58.5–86.8%)	91.7% (65.5–98.1%)	23.5 (13.9–39.8)
SPECT	3	316	83.7% (65.0–93.4%)	81.4% (66.0–92.9%)	15.6 (8.4–29.1)
Per lesion analysis					
CT	5	495	76.2% (67.6–83.1%)	91.0% (62.9–98.4%)	31.2 (21.4–45.5)
MRI	7	1746	90.6% (84.4–94.5%)	94.2% (77.2–98.4%)	66.2 (42.6–102.8)
PET/CT	4	428	91.8% (54.0–99.1%)	84.6% (55.2–96.1%)	18.9 (11.0–32.7)
BS	6	442	76.9% (51.8–91.2%)	52.2% (12.6–89.2%)	5.7 (4.2–7.7)
SPECT	4	322	92.3% (68.8–98.5%)	85.9% (70.5–93.9%)	29.6 (17.6–49.7)

**Fig. 3:**
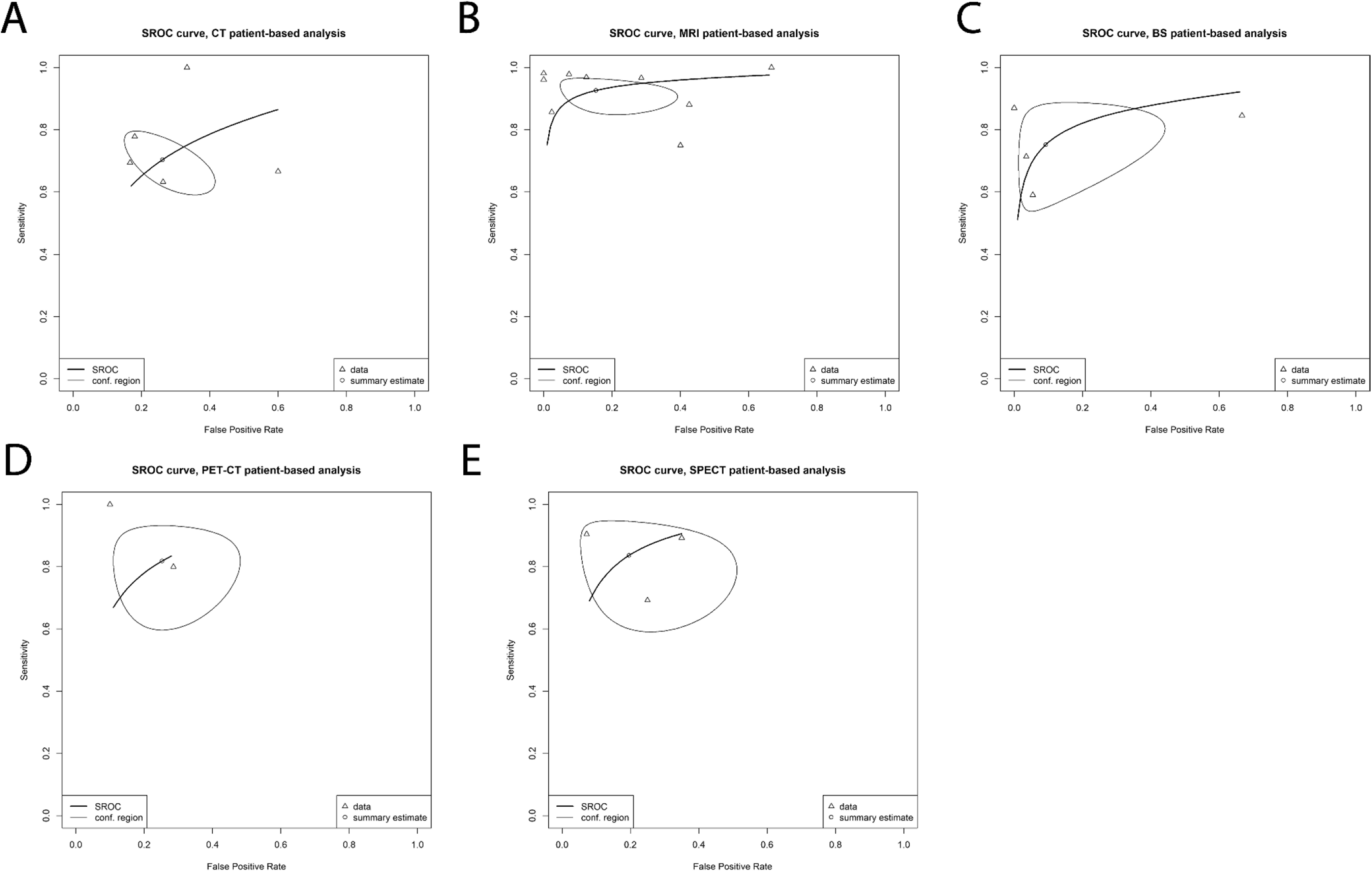
Patient-based analysis SROC curves. **A**: CT; **B**: MRI; **C**: BS; **D**: PET/CT; **E**: SPECT

MRI and BS had the highest DOR at 26.6 (95%CI 14.3–49.4) and 23.5 (95%CI 13.9–39.8). Heterogeneity was low for CT (*I*^2^ = 0%), MRI (*I*^*2*^ = 14.5%), FDG PET/CT (*I*^*2*^ = 17.7%), and BS (*I*^*2*^ = 39.9%). Analyses for SPECT had moderate heterogeneity (*I*^*2*^ = 62.8%). Lesion level SROC curves are provided in Fig. 3.

### Diagnostic performance of imaging modalities: lesion level

**Fig. 4:**
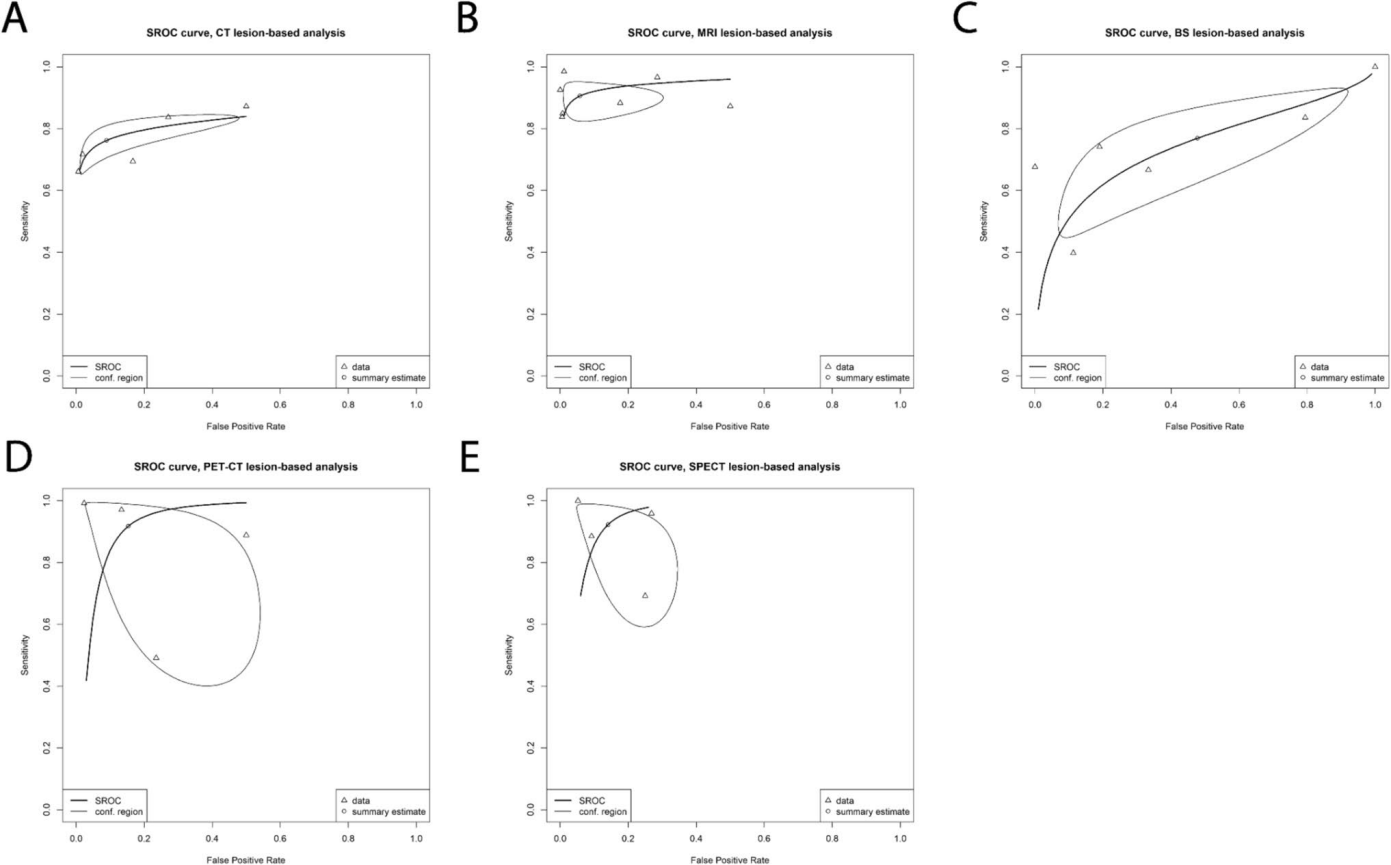
Lesion-based analysis SROC curves. **A**: CT; **B**: MRI; **C**: BS; **D**: PET/CT; **E**: SPECT

On a lesion level, 5 datasets were available for analysis for CT, 7 for MRI, 4 for FDG PET/CT, 6 for BS, and 4 for SPECT. Pooled sensitivities and specificities for CT were 76.2% (95%C: 67.6–83.1%) and 91.0% (62.9–98.4%), respectively. For MRI this was 90.6% (95%CI 84.4–94.5%) and 94.2% (95%CI 77.2–98.4%), respectively. For FDG PET/CT the pooled sensitivity and specificity were 91.8% (95%CI 54.0–99.1%) and 84.6% (95%CI 55.2–96.1%), respectively. The pooled sensitivity for SPECT was 92.3% (95%CI 68.8–98.5%) and specificity 85.9% (95%CI 70.5–93.9%). These metrics were much lower for BS with a sensitivity of 76.9% (95%CI 51.8–91.2%) and specificity of 52.2% (95%CI 12.6–89.2%). The highest DOR on a lesion level was for MRI (DOR: 66.2; 95%CI 42.6–102.8). Heterogeneity was low for CT (*I*^*2*^ = 0%), MRI (*I*^*2*^ = 42.8%), FDG PET/CT (*I*^*2*^ = 49.7%), and BS (*I*^*2*^ = 0%). Analyses for SPECT had moderate heterogeneity (*I*^*2*^ = 64%). Lesion level SROC curves are provided in Fig. 4.

### Subgroup analysis and publication bias

There was not enough data available to stratify analyses based on reference standard or tumor type in both patient- or lesion-based groups. Diagnostic accuracy results did not change for MRI after excluding one study with a field strength less than 1.5 Tesla (data not shown). Increased heterogeneity for SPECT could not be explained by tumor subtype, whereas other subgroups could not be explored due to the limited studies and lack of data. Deek Funnel plots for publication bias of each imaging modality on a patient and lesion level are provided in Supplementary Figure S1–S10, Appendix D. As the number of studies for each group was less than 10, Deek’s asymmetry test was not performed.

## Discussion

### Main findings

The diagnosis of spinal metastases has significant impact on treatment decisions and patient prognosis. In the present study, we assessed the diagnostic accuracy for spinal metastases detection in the staging setting of solid malignancies across a wide range of imaging modalities. Our results showed that MRI had the highest sensitivity on a patient level, whereas BS, MRI and SPECT had a high specificity on a per patient basis. CT and FDG PET/CT were the least accurate in the per patient analysis. On a lesion level, MRI, FDG PET/CT and SPECT had the highest sensitivity, whereas MRI and CT had the highest specificity. Conversely, BS had poor diagnostic accuracy for lesion detection. This was not surprising given that BS lacks precise anatomical lesion localization. Overall, MRI had the highest diagnostic accuracy in per patient and lesion analyses, and we observed low between study heterogeneity for all imaging modalities except SPECT.

In clinical practice, the standard workup of a patient with malignancy usually includes a thorax/abdomen CT, with or without radiographs. Depending on the primary tumor and curative treatment options, additional imaging can be performed with for example FDG PET-CT. In cancer patients, detecting spinal metastases is important before starting therapy, as curative treatment may be altered to palliative intent, due to the upstaging and worsened patient prognosis. In addition, spinal metastases detection is important to prevent (irreversible) neurological deficit. The Dutch National Guideline recommends MRI imaging when spinal metastases are suspected in patients with confirmed malignancy, usually when local back pain or other symptoms of neurological deficit are present [5]. However, specific literature of MRI performance in patients with suspected vertebral metastases and certain symptoms is lacking. Our findings suggest that the role of MRI needs to be reconsidered in staging of cancer patients subgroups with a high risk of spinal metastases. This includes certain patient characteristics (cancer patients with local back pain) or tumor characteristics (subtypes frequently metastasizing to the spine such as prostate, lung, and breast cancer). The added benefit of MRI needs to be carefully evaluated in prospective studies to assess the feasibility of its use and the risk of unnecessary additional imaging, especially in subgroups with low spinal metastases frequency (e.g. skin, esophagus, or colorectal cancer). Herein, the downsides of MRI imaging should also be considered [7]. These include increased costs and imaging times, as well as that MRI imaging may not be suitable for all patients, for example those with metal implants in the region of interest, specific pacemakers, or claustrophobia.

The studies in our meta-analysis had various reference standards including combinations of imaging, pathological confirmation, and/or clinical and imaging follow-up. It should be noted that in practice, diagnosing spinal metastases is not performed according to one imaging “golden standard”, but with pathological confirmation of metastasis from the spinal column or other anatomical location available for biopsy. Pathological confirmation may not always be performed in the case of extensive/diffuse disease or with histologically proven metastases in other locations, poor prognosis, or patients’ ability to undergo surgery or biopsy for example due to a poor performance status [45]. Moreover, if a patient has multiple spinal lesions, pathological confirmation is only obtained from one lesion. Likewise, clinical or imaging follow-up may not always feasible due to the poor overall survival in this population [46].

With regards to treatment decisions, CT is used for evaluating mechanical instability using the Spinal Instability Neoplastic Score (SINS) [47] and MRI for epidural spinal cord compression if alarm symptoms are present [48]. FDG PET/CT is more frequently used for spinal metastases detection, when spinal lesions are synchronously diagnosed during the staging of primary malignancies, and the choice for BS and SPECT is more dependent on specific tumor histology, for example more often in breast cancer patients.

Various factors may affect the diagnostic accuracy of imaging for spinal metastases detection. The performance across tumor subgroups should be taken into consideration when interpreting our results. For instance, it is well known that blastic type lesions (prostate, bladder, nasopharynx, bronchial cancer [8]) perform well on BS and SPECT [49]. On the other hand, lytic cancer types (breast, lung, kidney, oropharyngeal, thyroid, and melanoma cancer [8]) may be easier to identify on CT (including FDG PET/CT and SPECT). These classifications may vary by tumor type as mixed type lesions may also be present. Due to the limited data, we were not able to stratify our pooled analyses by lesion types.

Next to tumor characteristics, patient factors may also affect diagnostic performance. The presence of “red flags”, may increase the overall diagnostic accuracy of each modality [50]. For example, in a patient with malignancy history with a sudden increase in back pain or neurological symptoms, the likelihood of diagnosing spinal lesions may increase. Spinal lesions typically become more symptomatic with increasing size, which may result in bone destruction, vertebral body collapse, and complications like spinal cord or nerve root compression [4, 51]. To our knowledge, there are no studies comparing detection rates of spinal metastases in relation to clinical symptoms. MRI sequences should also be taken into consideration regarding diagnostic accuracy. Most commonly used.

We were not able to pool results based on MRI sequences as we had insufficient TP/FP/TN/FN data stratified by sequence. In practice, both T1w and T2w imaging with and without contrast administration are used for vertebral metastases detection. T2w fat-suppression sequences have been shown to be more sensitive than T1w and T2w imaging for vertebral metastases detection [7]. In addition, previous work found no significant differences between unenhanced and enhanced MRI sequences, other than increased sensitivity for unenhanced MRI on a patient level [52].

Specifically for FDG PET/CT, false negative findings may be more likely in spinal metastases with low glucose metabolism, or false positive findings in patients with inflammatory diseases [53].

Recent developments in medical imaging have prompted improvements in diagnostic accuracy and spinal metastases detection in the experimental setting. For example, dual energy spectral CT has shown promising results for differentiating between malignant and benign spinal lesions by means of novel multi-level calcium suppression techniques [26].

Other examples include sodium-fluoride PET in breast cancer spinal metastases [15], and fibroblast activation protein (FAP)-targeted radiotracers such as gallium-68 labeled FAP inhibitors ([68 Ga]Ga-FAPI) in bone metastases from lung cancer [54]. These methods may not be applicable for all tumor types, as for example, Ga-FAPI did not outperform conventional FDG PET/CT imaging across various cancer groups for bone metastases [55].

We found only two studies with individual data available on potassium fluoride (NaF) PETCT. One study reported results for NaF PET-CT on lesion level, for which the sensitivity was 96% and specificity 97% [15].

In practice, spinal metastases diagnosis is usually established with more than one imaging modality. The effect of combinations of imaging modalities on the diagnostic accuracy of spinal metastases would be an interesting aspect to explore. However, to the best of our knowledge, published studies on imaging combinations are lacking in the literature.

Strengths of our study include the analysis of the largest pooled patient population to date, the inclusion of the most recent literature, and we systematically studied FDG PET/CT for spinal metastasis detection, which has not been previously assessed [14]. Second, we performed detailed assessment of the methodological quality of the included studies with a validated instrument for diagnostic accuracy studies. Third, we were able to obtain additional (unpublished) data by contacting corresponding authors. The limitations of our meta-analysis should be mentioned. We were not able to perform detailed subgroup analyses due to the limited number of published studies. Second, estimates were pooled from both retrospective and prospective cohort study designs, for which the majority were retrospective in design. However, pooled estimates remained unchanged after excluding prospective design studies from our analyses. Finally, publication bias could not be quantitatively assessed.

In conclusion, in our study, we shed light on the performance of conventional and advanced imaging for spinal metastases detection. MRI had the highest diagnostic accuracy on a patient and lesion level for spinal metastases detection, suggesting a broader use in addition to routine staging CT, at least in patients at high risk and where detection of a spinal metastasis could alter therapy decisions. We discussed clinical and imaging aspects regarding spinal metastases detection in clinical practice, and highlighted the need for additional prospective studies evaluating combinations of imaging modalities and the inclusion of patient symptoms and factors.

## Supplementary Information

Below is the link to the electronic supplementary material.

Supplementary file1 (DOCX 1009 KB)

## Data Availability

Available upon reasonable request from the corresponding author.
