# Supplementary material for: Diagnostic accuracy of imaging modalities for detection of spinal metastases: a systematic review and meta-analysis
Source: Clin Transl Oncol. 2024 Oct 29;27(5):2316–26. doi: 10.1007/s12094-024-03765-1 (PMC12033096; doi:10.1007/s12094-024-03765-1)
Supplement: Supplementary file 1 — Supplementary file1 (DOCX 1009 KB) [file 12094_2024_3765_MOESM1_ESM.docx]

**APPENDIX A**

| **Section/topic** | **#** | **PRISMA-DTA Checklist Item** | **Reported on page #** |
| --- | --- | --- | --- |
| **TITLE / ABSTRACT** | | |  |
| Title | 1 | Identify the report as a systematic review (+/- meta-analysis) of diagnostic test accuracy (DTA) studies. | 1 |
| Abstract | 2 | Abstract: See PRISMA-DTA for abstracts. | 2 |
| **INTRODUCTION** | | |  |
| Rationale | 3 | Describe the rationale for the review in the context of what is already known. | 3 |
| Clinical role of index test | D1 | State the scientific and clinical background, including the intended use and clinical role of the index test, and if applicable, the rationale for minimally acceptable test accuracy (or minimum difference in accuracy for comparative design). | 3,4 |
| Objectives | 4 | Provide an explicit statement of question(s) being addressed in terms of participants, index test(s), and target condition(s). | 3,4 |
| **METHODS** | | |  |
| Protocol and registration | 5 | Indicate if a review protocol exists, if and where it can be accessed (e.g., Web address), and, if available, provide registration information including registration number. | 4 |
| Eligibility criteria | 6 | Specify study characteristics (participants, setting, index test(s), reference standard(s), target condition(s), and study design) and report characteristics (e.g., years considered, language, publication status) used as criteria for eligibility, giving rationale. | 4,5 |
| Information sources | 7 | Describe all information sources (e.g., databases with dates of coverage, contact with study authors to identify additional studies) in the search and date last searched. | 4 |
| Search | 8 | Present full search strategies for all electronic databases and other sources searched, including any limits used, such that they could be repeated. | 4, Appendix B |
| Study selection | 9 | State the process for selecting studies (i.e., screening, eligibility, included in systematic review, and, if applicable, included in the meta-analysis). | 4,5 |
| Data collection process | 10 | Describe method of data extraction from reports (e.g., piloted forms, independently, in duplicate) and any processes for obtaining and confirming data from investigators. | 5 |
| Definitions for data extraction | 11 | Provide definitions used in data extraction and classifications of target condition(s), index test(s), reference standard(s) and other characteristics (e.g. study design, clinical setting). | 4,5 |
| Risk of bias and applicability | 12 | Describe methods used for assessing risk of bias in individual studies and concerns regarding the applicability to the review question. | 5 |
| Diagnostic accuracy measures | 13 | State the principal diagnostic accuracy measure(s) reported (e.g. sensitivity, specificity) and state the unit of assessment (e.g. per-patient, per-lesion). | 5 |
| Synthesis of results | 14 | Describe methods of handling data, combining results of studies and describing variability between studies. This could include, but is not limited to: a) handling of multiple definitions of target condition. b) handling of multiple thresholds of test positivity, c) handling multiple index test readers, d) handling of indeterminate test results, e) grouping and comparing tests, f) handling of different reference standards | 5 |

| **Section/topic** | **#** | **PRISMA-DTA Checklist Item** | **Reported on page #** |
| --- | --- | --- | --- |
| Meta-analysis | D2 | Report the statistical methods used for meta-analyses, if performed. | 5 |
| Additional analyses | 16 | Describe methods of additional analyses (e.g., sensitivity or subgroup analyses, meta-regression), if done, indicating which were pre-specified. | 5 |
| **RESULTS** | | |  |
| Study selection | 17 | Provide numbers of studies screened, assessed for eligibility, included in the review (and included in meta-analysis, if applicable) with reasons for exclusions at each stage, ideally with a flow diagram. | 6, Figure 1 |
| Study characteristics | 18 | For each included study provide citations and present key characteristics including: a) participant characteristics (presentation, prior testing), b) clinical setting, c) study design, d) target condition definition, e) index test, f) reference standard, g) sample size, h) funding sources | 8, Table 1 |
| Risk of bias and applicability | 19 | Present evaluation of risk of bias and concerns regarding applicability for each study. | Figure 2 |
| Results of individual studies | 20 | For each analysis in each study (e.g. unique combination of index test, reference standard, and positivity threshold) report 2x2 data (TP, FP, FN, TN) with estimates of diagnostic accuracy and confidence intervals, ideally with a forest or receiver operator characteristic (ROC) plot. | Figure 3, Figure 4 |
| Synthesis of results | 21 | Describe test accuracy, including variability; if meta-analysis was done, include results and confidence intervals. | Table 2, 11, 15 |
| Additional analysis | 23 | Give results of additional analyses, if done (e.g., sensitivity or subgroup analyses, meta-regression; analysis of index test: failure rates, proportion of inconclusive results, adverse events). | 15 |
| **DISCUSSION** | | |  |
| Summary of evidence | 24 | Summarize the main findings including the strength of evidence. | 17-20 |
| Limitations | 25 | Discuss limitations from included studies (e.g. risk of bias and concerns regarding applicability) and from the review process (e.g. incomplete retrieval of identified research). | 20 |
| Conclusions | 26 | Provide a general interpretation of the results in the context of other evidence. Discuss implications for future research and clinical practice (e.g. the intended use and clinical role of the index test). | 20 |
| **FUNDING** | | |  |
| Funding | 27 | For the systematic review, describe the sources of funding and other support and the role of the funders. | 25 |

**APPENDIX B. Search Strategy**

1. **PUBMED**

| **Condition** | “spinal metastases” OR “vertebral metastases” OR “spinal metastasis” OR “vertebral metastases” OR (spine[MeSH] AND neoplasms[MeSH]) OR (spine AND cancer) |
| --- | --- |
| **Index test** | “Positron Emission Tomography Computed Tomography”[MeSH] OR ”Positron Emission Tomography Computed Tomography” OR  “PET-CT” OR “PET” OR “MRI” OR “magnetic resonance imaging”[MeSH] OR “magnetic resonance imaging” OR “Single Photon Emission Computed Tomography Computed Tomography”[MeSH] OR “Single Photon Emission Computed Tomography Computed Tomography” OR “SPECT” OR “CT” OR “Computed Tomography” OR “Tomography, X-Ray Computed”[MeSH] OR “Tomography, Spiral Computed”[MeSH] OR “Cone-Beam Computed Tomography”[MeSH] OR “Technetium Tc 99m Medronate” OR “99mTc-MDP” OR “Technetium Methylene Diphosphonate” OR “Technetium Tc 99m Methylenediphosphonate” OR “99mTc Methylene Diphosphonate” OR “Tc-99m Methylene Diphosphonate”OR “bone scan” OR “bone scanning” OR “bone scintigraphy” OR “bone scintiscanning” |
| **Diagnostic test accuracy terms** | “Sensitivity and Specificity”[MeSH] OR “Sensitivity” OR “Specificity” OR “PPV” OR “NPV” OR “Predictive Value of Tests”[MeSH] OR “Positive Predictive Value” OR “Positive Predictive Values” OR “Negative Predictive Value” OR “Negative Predictive Values” OR “positive likelihood ratio” OR “PLR” OR “negative likelihood ratio” OR “NLR” OR  “diagnostic odds ratio” OR “Diagnostic Accuracy” OR  “true positive” OR “TP” OR “false positive” OR “FP” OR “true negative” OR “TN” OR “false negative” OR “FN” |
| **Search:** | **Condition AND Index test AND Diagnostic test accuracy terms** |

1. **EMBASE:**

| **Condition** | (('spine'/exp OR spine) AND ('malignant neoplasm')) OR ‘spine metastasis’ OR ‘spinal metastases’ OR ‘vertebral metastases’ OR ‘spinal metastasis’ OR ‘vertebral metastases’ |
| --- | --- |
| **Index test** | 'positron emission tomography'/exp OR 'positron emission tomography' OR PET-CT OR ‘PET’ OR 'magnetic resonance imaging'/exp OR 'magnetic resonance imaging' OR (magnetic AND ('resonance'/exp OR resonance) AND ('imaging'/exp OR imaging)) OR MRI OR 'single photon emission computed tomography' OR SPECT OR 'computer assisted tomography' OR CT OR 'bone scintiscanning' OR ‘bone scan’ OR ‘bone scintigraphy’ |
| **Diagnostic test accuracy terms** | ‘Sensitivity’ OR ‘Specificity’ OR 'PPV' OR 'NPV' OR 'Positive Predictive Value' OR 'Positive Predictive Values' OR 'Negative Predictive Value' OR 'Negative Predictive Values' OR 'positive likelihood ratio' OR 'PLR' OR 'negative likelihood ratio' OR 'NLR' OR 'diagnostic odds ratio' OR 'Diagnostic Accuracy' OR 'true positive' OR 'TP' OR 'false positive' OR 'FP' OR 'true negative' OR 'TN' OR 'false negative' OR 'FN' |
| **Search:** | **Condition AND Index test AND Diagnostic test accuracy terms** |

1. **Web of Science:**

| **Condition** | “spinal metastases” OR “vertebral metastases” OR “spinal metastasis” OR “vertebral metastases” OR (spine AND cancer) |
| --- | --- |
| **Index test** | 'positron emission tomography' OR 'positron emission tomography' OR PET-CT OR ‘PET’ OR 'magnetic resonance imaging' OR MRI OR 'single photon emission computed tomography' OR SPECT OR 'computer tomography' OR CT OR 'bone scintiscanning' OR ‘bone scan’ OR ‘bone scintigraphy’ |
| **Diagnostic test accuracy terms** | ‘Sensitivity’ OR ‘Specificity’ OR 'PPV' OR 'NPV' OR 'Positive Predictive Value' OR 'Positive Predictive Values' OR 'Negative Predictive Value' OR 'Negative Predictive Values' OR 'positive likelihood ratio' OR 'PLR' OR 'negative likelihood ratio' OR 'NLR' OR 'diagnostic odds ratio' OR 'Diagnostic Accuracy' OR 'true positive' OR 'TP' OR 'false positive' OR 'FP' OR 'true negative' OR 'TN' OR 'false negative' OR 'FN' |
| **Search:** | **Condition AND Index test AND Diagnostic test accuracy terms** |

**Appendix C. Individual study risk of bias**

|  | **Risk of Bias** | | | | **Applicability Concerns** | | |
| --- | --- | --- | --- | --- | --- | --- | --- |
| **Study** | **Patient Selection** | **Index Test** | **Reference Standard** | **Flow and Timing** | **Patient Selection** | **Index Test** | **Reference Standard** |
| Cristo Santos 2023 | 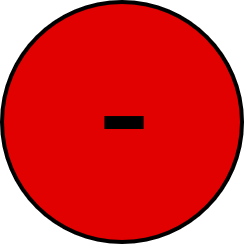 | 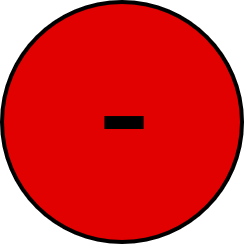 | 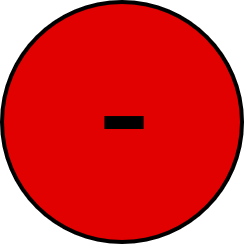 | 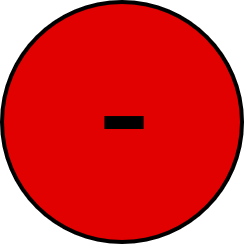 | 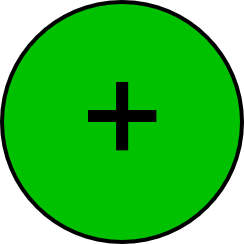 | 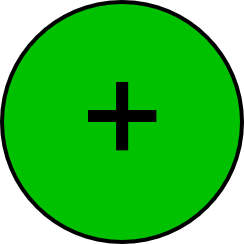 | 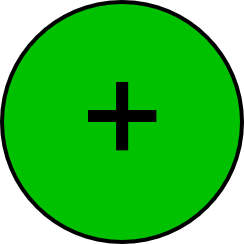 |
| Panagiotidis 2023 | 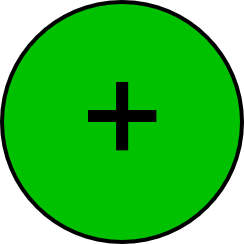 | 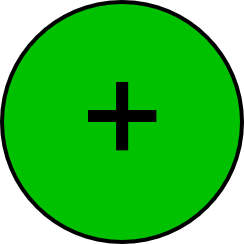 | 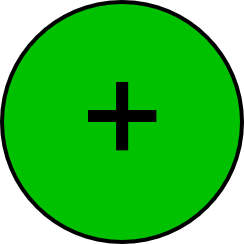 | 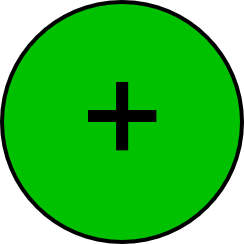 | 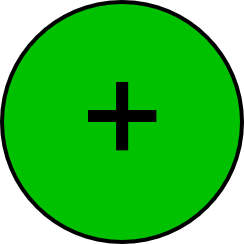 | 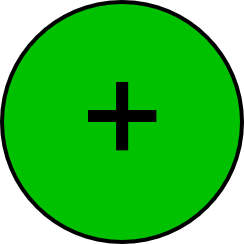 | 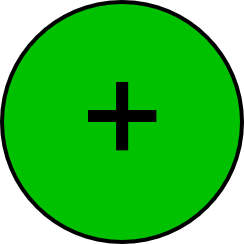 |
| Zarad 2023 | 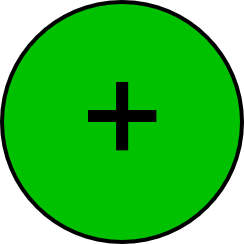 | 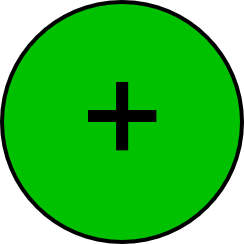 | 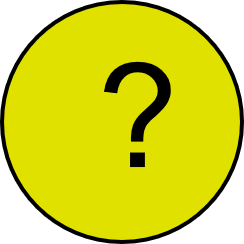 | 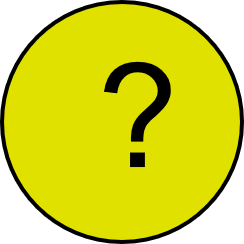 | 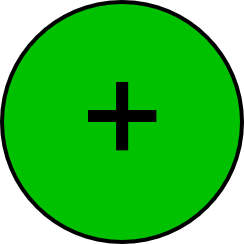 | 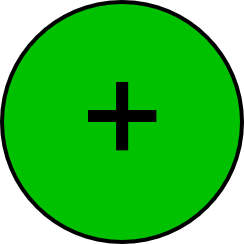 | 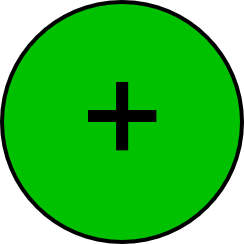 |
| Qin 2022 | 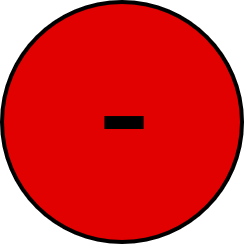 | 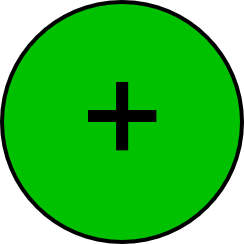 | 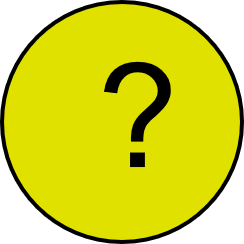 | 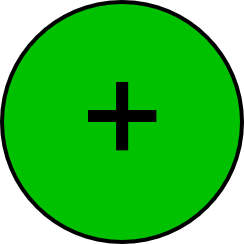 | 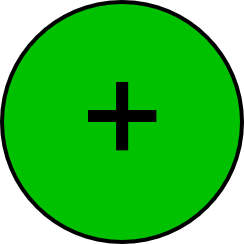 | 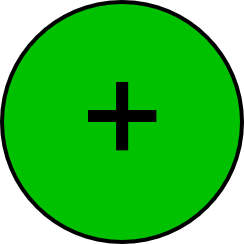 | 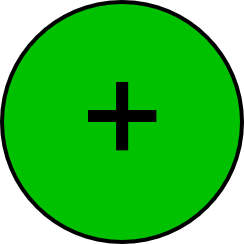 |
| Jung 2021 | 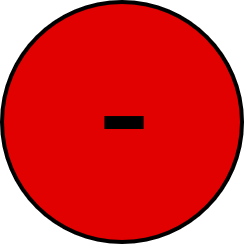 | 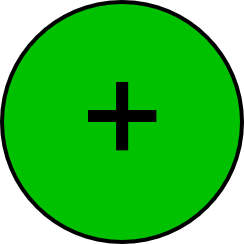 | 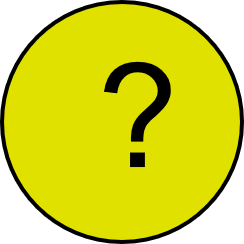 | 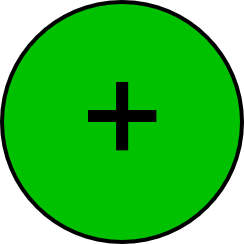 | 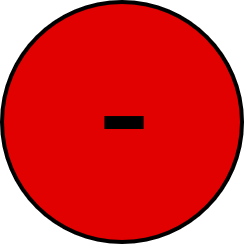 | 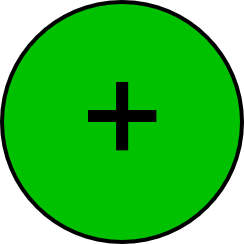 | 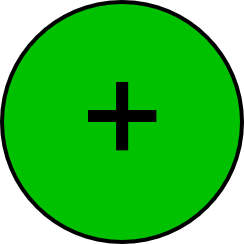 |
| Liu 2020 | 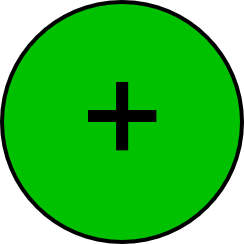 | 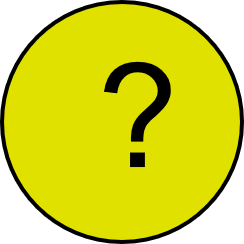 | 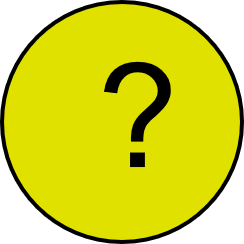 | 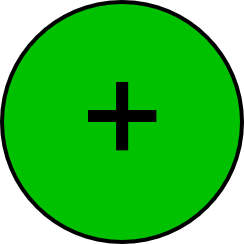 | 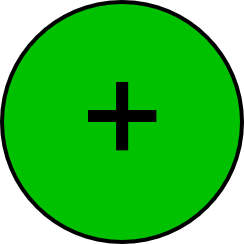 | 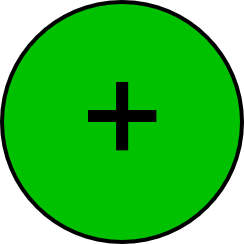 | 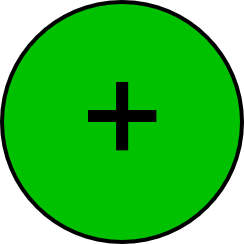 |
| Abdullayev 2019 | 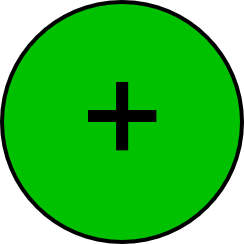 | 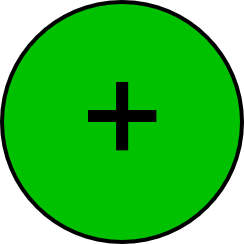 | 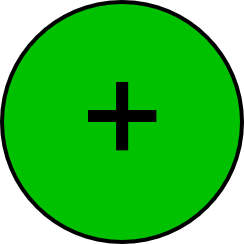 | 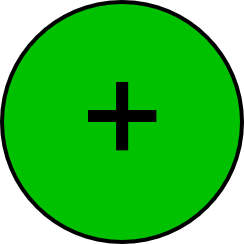 | 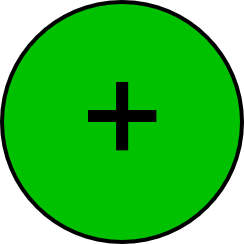 | 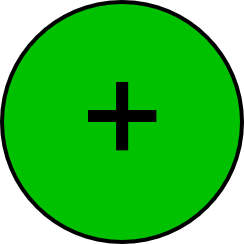 | 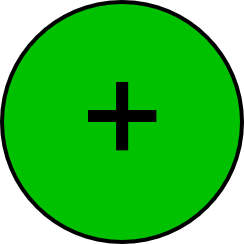 |
| Mavriopoulou 2018 | 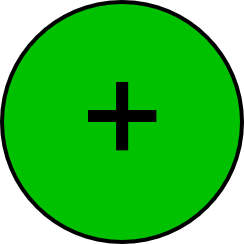 | 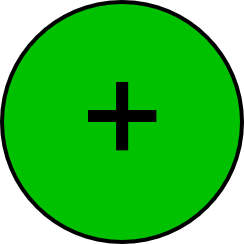 | 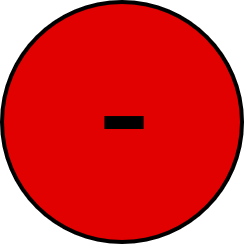 | 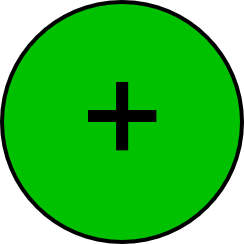 | 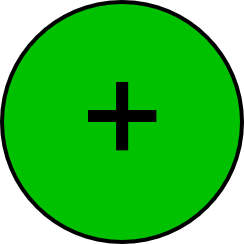 | 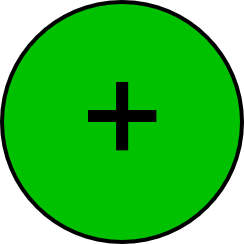 | 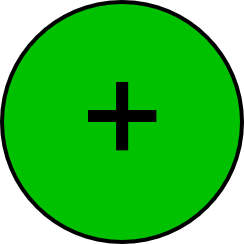 |
| Maeder 2018 | 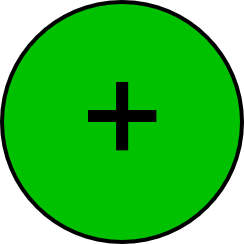 | 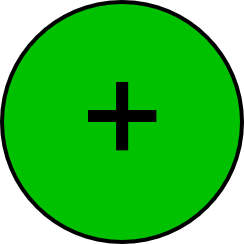 | 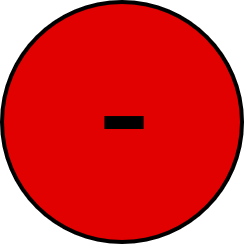 | 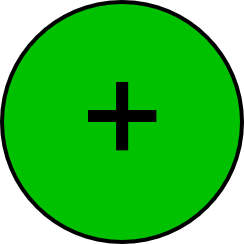 | 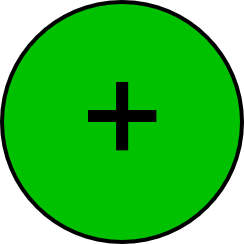 | 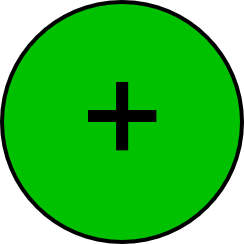 | 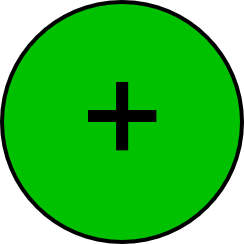 |
| Iwano 2017 | 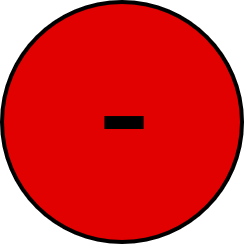 | 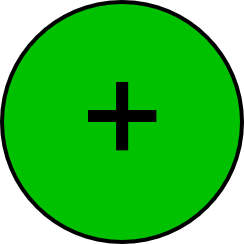 | 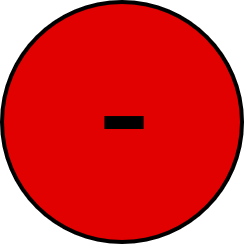 | 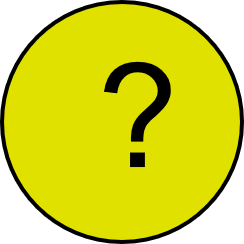 | 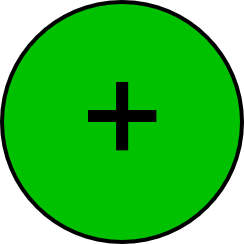 | 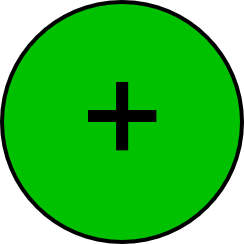 | 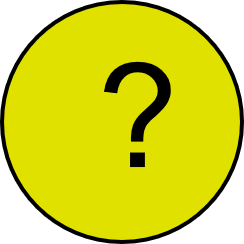 |
| Park 2017 | 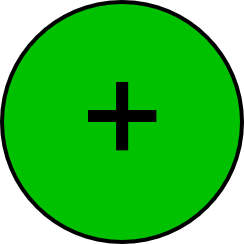 | 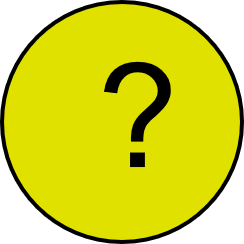 | 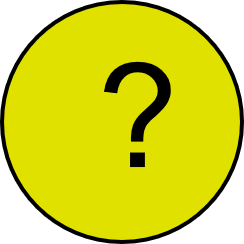 | 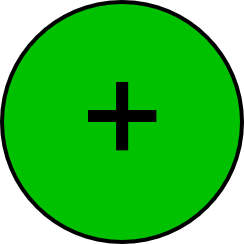 | 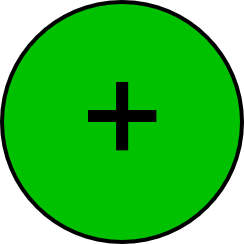 | 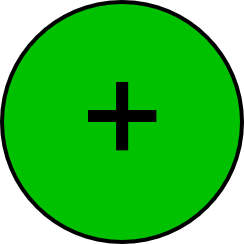 | 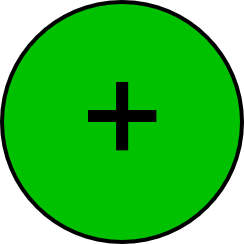 |
| Lange 2016 | 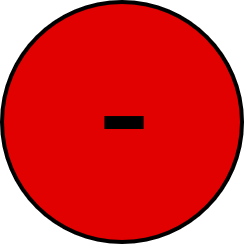 | 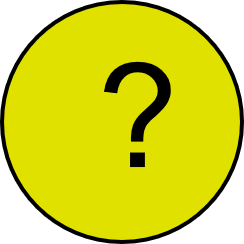 | 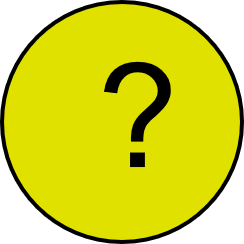 | 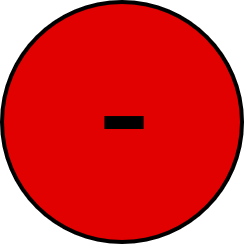 | 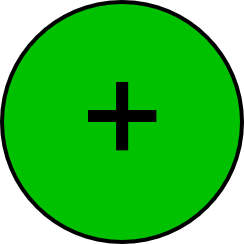 | 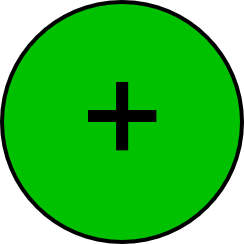 | 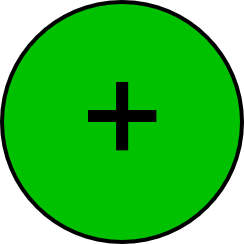 |
| Zidan 2014 | 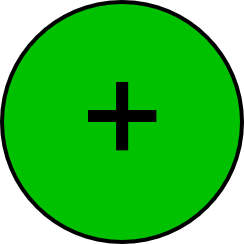 | 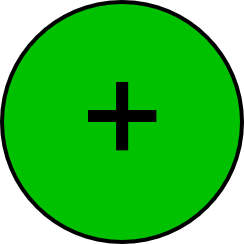 | 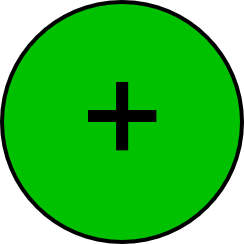 | 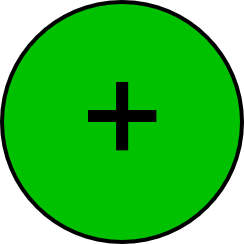 | 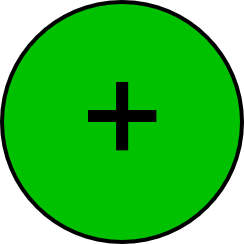 | 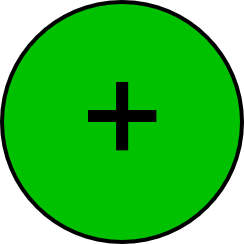 | 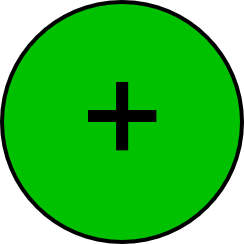 |
| Uchida 2013 | 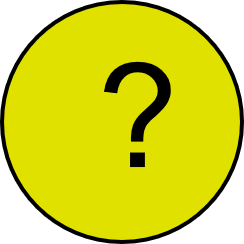 | 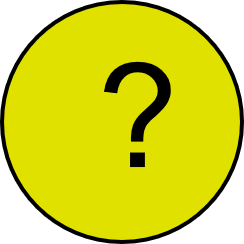 | 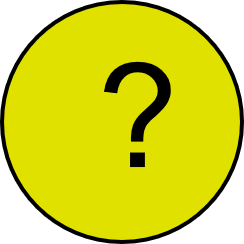 | 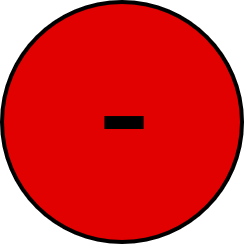 | 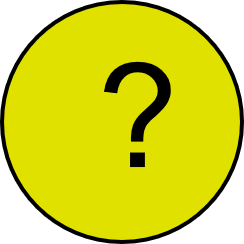 | 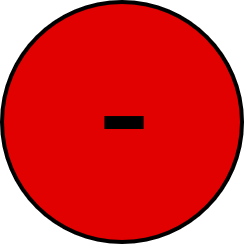 | 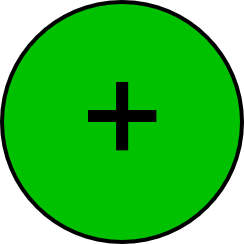 |
| Venkitaraman 2009 | 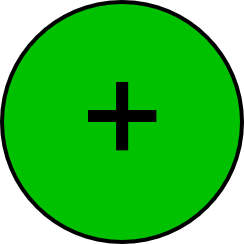 | 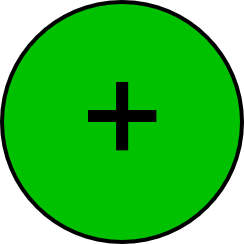 |  |  |  |  |  |
| Buhmann 2009 |  |  |  |  |  |  |  |
| Nozaki 2008 |  |  |  |  |  |  |  |
| Altehoefer 2001 |  |  |  |  |  |  |  |
| Han 1998 |  |  |  |  |  |  |  |
| Petrén-Mallmin 1993 |  |  |  |  |  |  |  |
| Savelli 2001 |  |  |  |  |  |  |  |
| Ohno 2003 |  |  |  |  |  |  |  |
| Hahn 2018 |  |  |  |  |  |  |  |
| Wafaie 2013 |  |  |  |  |  |  |  |
| Oh 2005 |  |  |  |  |  |  |  |

Red = high risk; Green = low risk; Yellow = unclear risk

**Appendix D. Deek Funnel plots for each modality on a patient and lesion level**

Figure S1. CT patient level

Figure S2. CT lesion level

Figure S3. MRI patient level

Figure S4. MRI lesion level

Figure S5. PET-CT patient level

Figure S6. PET-CT lesion level

Figure S7. Bone Scintigraphy patient level

Figure S8. Bone Scintigraphy lesion level

Figure S9. SPECT patient level

Figure S10. SPECT lesion level
